# Thermal grill conditioning: Effect on contact heat evoked potentials

**DOI:** 10.1038/srep40007

**Published:** 2017-01-12

**Authors:** Catherine R. Jutzeler, Freda M. Warner, Johann Wanek, Armin Curt, John L. K. Kramer

**Affiliations:** 1 Spinal Cord Injury Center, University Hospital Balgrist, University of Zurich, Zurich, Switzerland; 2 ICORD, University of British Columbia, Vancouver, BC, Canada; 3 School of Kinesiology, University of British Columbia, Vancouver, BC, Canada

## Abstract

The ‘thermal grill illusion’ (TGI) is a unique cutaneous sensation of unpleasantness, induced through the application of interlacing warm and cool stimuli. While previous studies have investigated optimal parameters and subject characteristics to evoke the illusion, our aim was to examine the modulating effect as a conditioning stimulus. A total of 28 healthy control individuals underwent three testing sessions on separate days. Briefly, 15 contact heat stimuli were delivered to the right hand dorsum, while the left palmar side of the hand was being conditioned with either neutral (32 °C), cool (20 °C), warm (40 °C), or TGI (20/40 °C). Rating of perception (numeric rating scale: 0–10) and evoked potentials (i.e., N1 and N2P2 potentials) to noxious contact heat stimuli were assessed. While cool and warm conditioning decreased cortical responses to noxious heat, TGI conditioning increased evoked potential amplitude (N1 and N2P2). In line with other modalities of unpleasant conditioning (e.g., sound, visual, and olfactory stimulation), cortical and possibly sub-cortical modulation may underlie the facilitation of contact heat evoked potentials.

In 1896, Torsten Thunberg first described the paradoxical sensation of heat in response to interlaced warm and cool stimulation. Since these seminal observations, the “thermal grill illusion” (TGI) has been described extensively using various modern stimulating devices. Of defining characteristics, the TGI is reported as a uniquely “unpleasant” somatosensory experience^1^,^2^,^3^,^4^,^5^,^6^,^7^,^8^. Central disinhibition related to persistent activation in polymodal nociceptive spinothalamic cells, coupled with reductions of activity in innocuous thermoreceptive spinothalamic cells has been proposed as underlying the unpleasantness of the TGI^6^,^9^,^10^. Neuroimaging investigations have demonstrated activation in prominent brain areas, including the anterior cingulate cortex (ACC)^11^. Activation in the ACC during the TGI is notable because this area has been implicated in processing unpleasant visual, auditory, and olfactory stimuli^12^,^13^,^14^,^15^.

Investigating the conditioning effects of afferent stimuli on responses to noxious input has generated a wealth of knowledge regarding endogenous pathways modulating pain. The notion of “anti-nociception” has been largely explored using conditioning *somatosensory* input, which is typically perceived as both painful and unpleasant^16^,^17^,^18^,^19^. In contrast, “pro-nociception” has been demonstrated using unpleasant auditory, visual, and olfactory conditioning (i.e., *special senses*)^12^,^14^,^20^,^21^,^22^,^23^,^24^. To our knowledge, no studies have bridged anti- and pro-nociception using a purely unpleasant somatosensory conditioning stimulus (i.e., not perceived as painful).

The objective of the current study was to address the heterotopic conditioning effect of an unpleasant somatosensory afferent stimulus (i.e., interlaced hot and cool bars) on responses to noxious contact heat stimulation. In line with other forms of unpleasant sensory conditioning, we hypothesized that the TGI would result in increased responses to noxious afferent stimulation. To explore changes in cortical activity, the conditioning effect of the TGI was examined using contact heat evoked potentials (CHEPs).

## Material and Methods

### Subjects

A total of 32 healthy control individuals (31.2 ± 6.9 years; gender: 13 female, 19 male) were recruited. Inclusion criteria were (1) age between 18–40 years, (2) being naïve to TGI and CHEPs, and (3) fluent in English or German. Exclusion criteria comprised pregnancy, intake of any medication (except birth control), and previous participation in a TGI or CHEPs study. All participants provided written informed consent and all procedures described below were in accordance with the Declaration of Helsinki and approved by the local ethics board ‘Kantonale Ethikkommission Zürich, KEK’ (ref. number: EK-04/2006; cinicaltrial.gov number: NCT02138344).

### Study protocol

Prior to the commencement of the study, all individuals were interviewed to measure pain catastrophizing using the German or English version of the pain catastrophizing scale (PCS)^25^. The PCS investigates individuals catastrophic thinking related to painful experiences. The overall reliability and validity of the PCS has been demonstrated in earlier studies^26^,^27^,^28^,^29^. Previous studies have demonstrated significant correlations with endogenous pain modulation and PCS^30^,^31^,^32^, and the TGI and PCS^33^.

**Figure 1:**
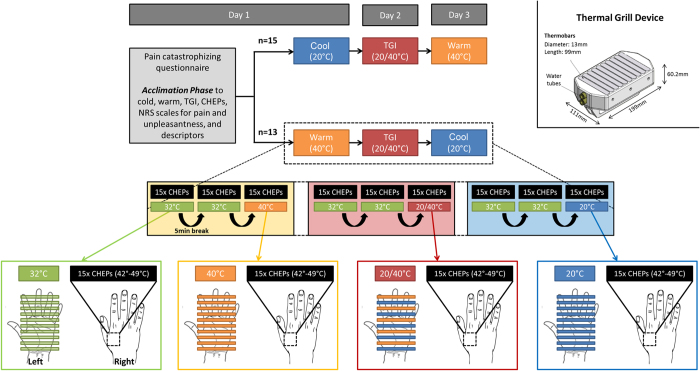
Experimental design. The study comprised of three different testing days differing only by the conditioning modalities applied. On the first day, all individuals were familiarized with the stimulation devices (CHEPS Stimulator (Pathway, Medoc, RamatYishai, Israel) and customized Thermal Grill device (http://www.sms.hest.ethz.ch/; Engineering Department)) and the temperatures (i.e., neutral, warm, cool, and interlaced cool/warm) that would be used as conditioning modalities. Employing the thermal grill device, all bars were set to either 32 °C (neutral), 20 °C (cool), 40 °C (warm), or alternating 20/40 °C (i.e., the TGI). The individuals were also exposed to three contact heat stimulations delivered by the CHEPS thermode. A 30-minute break followed the familiarization phase upon the commencement of the first experimental session to avoid thermal carry-over effects. The experiment consisted of three measurement blocks of combined conditioning and contact heat stimulation separated by breaks of 5 minutes. For the first two blocks, neutral (32 °C) was chosen as the conditioning modality, while either warm (W), cool (C), or interlaced cool/warm (TGI) (i.e., pseudo-randomized order over the three days: C/TGI/W or W/TGI/C) was presented in the third block. Each block was initiated with a 30 s exposure to the conditioning modality (i.e., neutral, cool, warm, or interlaced cool/warm) by placing the left hand on the thermal grill. Subsequently, individuals were instructed to keep the left hand on the thermal grill device, to fix on a point on the ceiling with their eyes, and to remain relaxed and quiet while recording of the 15 contact heat evoked potential stimulations (CHEPS) applied on the contralateral right hand. All contact heat stimulations were made from a baseline temperature of 42 °C to a peak temperature of 52 °C. The nominal heating rate was 70 °C/s and the cooling rate was 40 °C/s.

The study consisted of three different testing days differing only by the conditioning stimulation applied (Fig. 1). On the first day, all individuals were familiarized with the stimulation devices (CHEPS Stimulator and Thermal Grill device) and the temperatures (i.e., neutral, warm, cool, and interlaced cool/warm) that would be used for conditioning. All stimulation conditions were delivered using an identical device. Bars were set to either 32 °C (neutral), 20 °C (cool), 40 °C (warm), or alternating 20/40 °C (i.e., the TGI). Each condition was presented to the individuals for 30 s followed by a short interview to assess whether the conditions were perceived as unpleasant or painful (yes/no). If, and only if, a subject indicated unpleasantness or pain, they were then asked to rate the pain/unpleasantness by means of a numeric rating scale ranging from 0 (not unpleasant/painful at all) to 10 (very unpleasant or most unbearable pain). We believe that this is an important distinction from studies that cue subjects to rate unpleasantness/pain without first identifying if the sensation was unpleasant/painful^34^. Independent of their reported perception, all individuals were asked to describe the various modalities from a list of descriptors (e.g., warm, burning, cool, freezing, sharp, itching, and unpleasant). The goal of the familiarization phase was to reduce the novelty factor and potential anxiety during the experiment. To avoid thermal carry-over effects, a 30-minute break followed the familiarization phase before commencing the first conditioning experiment. Half of the subjects were randomized to warm (all bars 40 °C) and the other half were randomized to cool (all bars 20 °C). On the second experimental day, all subjects underwent TGI conditioning. On day 3, subjects crossed-over into warm and cool conditions.

During the experiment, subjects were lying in a supine position with eyes open. The experiment consisted of three measurement blocks of combined conditioning and contact heat stimulation separated by 5-minute rest breaks. The first two blocks of contact heat stimulation were delivered to the right hand during neutral (32 °C) conditioning. In a previous study, we observed significant reductions in CHEPs following repetitive stimulation in the same cutaneous area^35^. Based on these observations, we applied two neutral conditions to establish a stable CHEPs baseline from which to measure changes during conditioning. Warm, cool, or interlaced cool/warm (TGI) was presented in the third block to the left hand. Each block was initiated with a 30 s exposure to the conditioning modality (i.e., neutral, cool, warm, or interlaced cool/warm) by placing the left hand on the thermal grill stimulator. Subjects were instructed to keep the left hand on the thermal grill device, to fix on a point on the ceiling with their eyes, and to remain relaxed and quiet while recording of the CHEPs. Examiners noted if subjects withdrew their hand entirely from the thermal grill for all of the conditions. A total of 15 contact heat stimuli were delivered at an interpulse interval of 8 to 12 seconds. Individuals were asked to rate the perceived intensity of each stimulus from 0 (no pain) to 10 (most unbearable pain) two seconds after stimulation in response to an audio cue. To reduce receptor fatigue or sensitization by overheating of the skin^36^, the thermode was slightly repositioned following each stimulus within the right C6 dermatome (an area of approximately 4 × 4 cm).

### Stimulating device and recording

Responses to noxious stimuli were examined using a contact heat stimulator (Pathway, Medoc, RamatYishai, Israel). The thermode surface (diameter: 27 mm) consists of a heating thermo-foil covered with a layer of thermos-conductive plastic. The nominal heating rate of this device is 70 °C/s (thermo-foil), with a cooling rate of 40 °C/s (peltier element). From peak temperature, the thermode immediately returned to baseline. All measurements of contact heat stimulation were made from a baseline temperature of 42 °C to a peak temperature of 52 °C^37^, yielding a pulse duration of approximately 393 ms (baseline to peak temperature = 143 ms, peak temperature to baseline = 250 ms).

CHEPs were recorded with 9 mm Ag/AgCl surface disc electrodes filled with conductive adhesive gel. Scalp recording sites were prepared with Nuprep (D.O. Weaver & Co. Aurora, CO) and alcohol. In brief, CHEPs are an electrophysiological approach to explore cortical responses to A-delta afferents activated by rapid heat stimuli delivered peripherally. The early CHEPs component (N1) represents a direct measure of nociceptive input at the primary somatosensory cortex^38^,^39^. The late waveform (N2P2) reflects later stage processing of the noxious stimulus in the anterior cingulate gyrus and secondary somatosensory cortex^38^,^39^. Cortical recording electrodes were positioned according to the International 10–20 system based on available guidelines^40^. N2P2 was acquired from an active vertex recording electrode (Cz) referenced to linked earlobes (A1-A2)^41^,^42^,^43^,^44^,^45^,^46^. A contralateral temporal active recording electrode (Tc) referenced to Fz was used to acquire N1 as described previously^37^. All signals were sampled at 2000 Hz using a preamplifier (20000x, bandpass filter 1–300 Hz, ALEA Solutions, Zurich, Switzerland). Data were recorded with 100 ms pre-trigger and a one second post-trigger in a LabView based program (V1.43 CHEP, ALEA Solutions, Zurich, Switzerland). The N2P2 and N1 waveforms were visually detected based on the average of the 15-recorded trials.

### Thermal Grill device

A customized thermal grill device was built by Sensory-motor system laboratory at the Swiss Federal Institute of Technology (ETH) Zurich (http://www.sms.hest.ethz.ch/; Engineering Department). The device is equipped with 10 bars (bar dimensions: length 99 mm, diameter 13 mm) each consisting of three Peltier elements, all of which capable of dynamic cooling and heating within a minimum range between 18 and 42 °C. All thermoelectric bars were individually controlled by LabVIEW (LV) software (LV2010, Austin, TX, USA). The software was developed at the Spinal Cord Injury Center (Balgrist University Hospital, Zurich, Switzerland).

### Statistical analysis

The statistical analysis focused on addressing changes in CHEP outcomes (rating and N1/N2P2 amplitude) from the neutral condition to warm, cool, and interlaced cool/warm conditioning. “Neutral” CHEPs outcomes were examined to determine their distribution (i.e., normality). Based on an evaluation of skewness and kurtosis parametric statistics were planned. An unstructured linear mixed effects model was applied to examine the main effect of repeated stimulation (second neutral and conditioned) and interaction effect with different condition modalities (warm, cool, interlaced warm and cool). The advantage of the LMM is that one can account for missing data when analyzing longitudinal data^47^. From this model, we were most interested to determine if conditioning differentially impacted CHEPs outcomes (i.e., interaction effect). In the case of a significant interaction effect, pair-wise comparisons (t-tests) were planned to examine changes in the corresponding CHEPs outcome.

We next considered the relationship between rating of unpleasantness and changes in CHEP outcomes during TGI conditioning. Changes in N1/N2P2 amplitude and pain rating (from the second neutral condition to TGI conditioning) were examined using separate analysis of variance (ANOVA) for N1 and N2P2. The effect of unpleasantness was first examined by way of dichotomizing subjects as “responders” and “non-responders”. TGI responders were defined as any subject that reported the TGI as more unpleasant than warm and cool conditions. Non-responders perceived the TGI as equally or less unpleasant than warm or cool conditioning. Responders/Non-responders was treated as a between subject factor in the models. In order to further explore the relationship between perception and changes in CHEPs outcomes, a second ANOVA explored the effect of TGI unpleasantness as a covariate.

All statistical testing was performed in SPSS (V. 23) and statistical significance was set at p < 0.05.

## Results

### Subjects

Out of 32 healthy subjects enrolled in the study 4 had to be excluded due to: 1) technical problems during data acquisition (n = 2) and 2) intolerance of the contact heat stimuli applied (n = 2). The remaining 28 subjects comprised of 11 men and 17 women (mean age: 29.4 ± 6.3 years). The mean score of the PCS questionnaire was 11.9 ± 7.7. Participants’ characteristics are summarized in Table 1.

### Perception of neutral, cool, warm, and interlaced cool/warm

During exposure to the neutral condition (32 °C), no unpleasantness or pain was reported. The cool modality was reported to be unpleasant by five individuals (NRS_unpleasantness_ = 3.2 ± 2.8) and painful by one individual (NRS_pain_ = 2 ± 0). In response to warm conditioning, four individuals reported unpleasantness (NRS_unpleasantness_ = 1 ± 0) and one pain (NRS_pain_ = 1). The descriptors used for cool stimulation were cool (n = 18), cold (n = 9), and freezing (n = 1). The warm modality was described as warm (27) and burning (1). A total of 13 individuals indicated the interlaced application of cool/warm to be unpleasant (NRS_unpleasantness_ = 3.7 ± 2.1) (summarized in Table 1). The descriptors used by the individuals during the TGI were unpleasant (n = 13), weird (n = 6), confusing (n = 5), hot (n = 4), burning (n = 4), painful (n = 3), and irritating (n = 2). Fourteen individuals spontaneously withdrew their hand in relation to the TGI. All individuals who initially withdraw were able to return their hand for measurement of CHEPs. None of the subjects withdrew their hand to warm or cool conditions. Overall, 10 subjects reported higher unpleasantness during the TGI compared to warm and/or cool conditions (i.e., TGI responders).

### Effects of heterotopic conditioning on CHEPs (warm, cool, TGI)

Summary N1 and N2P2 amplitudes, latencies for N1 as well as N2 and P2, and pain ratings of three different conditions (i.e., warm, cold, and TGI) are presented in Table 2.

**Figure 2:**
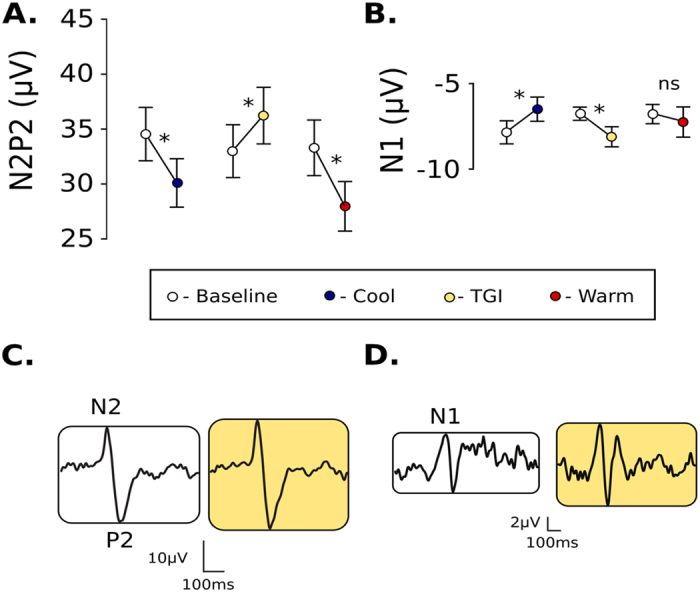
Effect of the condition modalities (cool, warm, TGI) on cortical responses to noxious heat stimulation (N2P2 and N1 amplitudes). (**A**) N2P2 and (**B**) N1 changes in amplitude during cool, warm, and TGI conditioning (+/−standard error). Only TGI conditioning resulted in facilitation of cortical responses to noxious heat stimulation. Averaged N2P2 and N1 waveforms (**C,D**), demonstrating facilitation of CHEPs during TGI conditioning.

N2P2 amplitude was significantly affected by the heterotopic conditioning stimulation (main effect: df = 23.7, F = 23.70, p < 0.001), which depended on the conditioning modality (interaction effect: df = 27.3, F = 9.16, p < 0.001). Significant decreases were observed between during warm (t = 6.02, p < 0.001) and cool conditioning (t = 4.97, p < 0.001). In contrast, N2P2 amplitude was significantly *increased* during interlaced warm and cool conditioning (t = 3.05, p = 0.005) (Fig. 2).

There was no significant effect of repeated stimulation on N1 amplitude (main effect: df = 20.0, F = 0.02, p = 0.89). The modulation of N1 amplitude was, however, dependent on the conditioning modality (interaction effect: df = 19.4, F = 8.32, p = 0.002). During cool conditioning, N1 amplitude significantly decreased (t = 2.52, p = 0.02). While warm conditioning had no significant effect (t = 0.14, p = 0.892), TGI conditioning significantly *increased* N1 amplitude (t = 2.54, p = 0.021) (Fig. 2). There was no main effect of stimulation or interaction effect for ratings to CHEP stimulation.

### Relationship between TGI unpleasantness and changes in N1/N2P2 amplitude

“Responders” and “non-responders” yielded similar changes in N1 (df = 18, F = 0.017, p = 0.899) and N2P2 amplitudes (df = 26, F = 0.911, p = 0.349). As a covariate, there was no effect of “unpleasantness” rating on N1 (df = 18, F = 1.148, p = 0.298) or N2P2 amplitude (df = 26, F = 2.721, p = 0.111).

## Discussion

In the 100-year history of the TGI, research has focused primarily on establishing the quality of sensation unmasked by stimulating with interlaced, innocuous warm and cool stimuli^3^,^5^,^6^,^48^. Others have examined factors that influence the perception of unpleasantness resulting from the thermal grill (e.g., spatial representation)^7^,^49^,^50^,^51^. In the present study, we applied the TGI as a heterotopic conditioning stimulus and revealed the discrete facilitation of cortical responses to noxious afferent input.

Several lines of converging evidence indicate an important distinction between early and late CHEP waveforms, whereby N1 represents a direct measure of nociceptive input and activation in the primary somatosensory cortex, and N2 and P2 represents later stage processing in the ACC and secondary somatosensory cortex^38^,^52^,^53^,^54^. Based on this physiological distinction, CHEPs have the potential to differentiate modulation occurring at different levels along the neuroaxis. Cortical modulation has been proposed based on reduced N2P2 amplitude in the absence of N1 changes (e.g., placebo analgesia)^55^. In contrast, changes in N1 and N2P2 amplitude have been purported as evidence of subcortical modulation (e.g., caloric vestibular stimulation and touch)^56^,^57^.

Based on increases in both N1 and N2P2 amplitude during TGI conditioning, it is intriguing to consider that responses to noxious heat were modulated sub-cortically. One potential explanation is that modulation occurs at the spinal level. Previous investigations have demonstrated increases in nociception during unpleasant auditory^22^,^58^, visual^12^,^15^,^23^,^24^,^59^, and olfactory conditioning^60^. Closely related to our observations, unpleasant visual stimuli have been shown to increase perception and the amplitude of electrical evoked pain related potentials^23^. In line with facilitation of N1 and subcortical modulation, the nociceptive flexion reflex is enhanced by unpleasant visual conditioning^12^,^24^,^59^,^61^,^62^,^63^. The facilitation of unpleasant visual stimuli at the spinal level has been attributed to activation in the amygdala, which projects to structures involved in descending control^64^.

Interestingly, however, TGI related unpleasantness did not have a significant effect on the magnitude of changes in CHEPs – that is, TGI responders demonstrate increases comparable to non-responders. In our study, 10/28 healthy subjects described the TGI as more unpleasant than warm and cool conditioning (responders = 36%). This number is somewhat lower than previous studies^3^, and may be related to differences in stimulation protocol (e.g., 40/16 °C versus 40/20 °C), how unpleasantness was assessed, and/or variable pressures that subjects manually applied to the thermal grill.

The lack of a relationship between unpleasantness and the facilitation of CHEPs may be a function of underpowered statistics (i.e., only 10 subjects reporting TGI as distinctly unpleasant). More generally, problems may arise from defining “responders”. In the current study, a responder had to identify the TGI as more unpleasant than either warm or cool conditions. Some subjects did not report TGI as “unpleasant” but still physically removed their hand during presentation with interlaced warm and cool bars. This reflexive motor response could be construed, at some level, as evidence of discomfort or unpleasantness associated with the thermal grill. Alternatively, withdrawal (and other descriptors commonly applied, e.g., “weird”) may reflect the TGI as a “metaesthesic” sensation^65^ – that is, sensation before pain, which is sometimes (but not always) described as unpleasant^2^. At the core of the problem are confounding variables that contribute to whether an individual reports the thermal grill as unpleasant. Regardless, the ubiquitous facilitation of CHEPs suggests that processes (e.g., central disinhibition) underlying the TGI are independent of subjective perception.

### Future directions and limitations

The current study was designed with all subjects undergoing the TGI on the second day of testing. Subjects were randomly assigned to cool or warm for the first session, and crossed into the other condition at the third session. This design was intended to overcome the problem that varying the time from the original familiarization period could influence future responses to the TGI. One potential problem with this approach is that the conditioning effect of TGI on CHEPs may depend on whether the warm or the cool condition came first. We also cannot rule out that the “TGI-effect” on CHEPs was related to being tested on day 2.

Another limitation is that we did not specifically assay changes in arousal. Previous studies demonstrate a strong attention/vigilance component for N2P2, and, to a lesser extent, N1^39^,^66^,^67^,^68^. Increased arousal related to the TGI may then explain, in part, facilitated CHEPs. Increased arousal has been reported in response to the TGI^34^. In this previous study, greater arousal was related to more unpleasantness^34^. In order to explain the facilitation of CHEPs, because there was no relationship between unpleasantness and increases in N1 and N2P2 amplitude, the TGI would have had to increase arousal in “non-responders”. Otherwise, the effect of arousal on CHEPs would be bundled into the effect of unpleasantness. Additional investigation is warranted to clarify the effect of the TGI on arousal. Examining cortical responses to other forms of non-noxious stimulation (e.g., electrical) should also be considered to determine if the effect of TGI is specific to noxious stimulation.

## Conclusion

In summary, this is the first study providing evidence that heterotopic TGI conditioning facilitates nociceptive evoked potentials. Based on the pattern of facilitation (i.e., both N1 and N2P2), the TGI modulated CHEPs sub-cortically (e.g., spinal cord). Facilitation occurred independently of reported unpleasantness, suggesting that the TGI has a similar pro-nociceptive effect regardless of how it is perceived. Future studies are warranted to further examine the somatosensory conditioning effects of the TGI on cortical responses to afferent stimulation.

## Additional Information

**How to cite this article**: Jutzeler, C. R. *et al*. Thermal grill conditioning: Effect on contact heat evoked potentials. *Sci. Rep.* **7**, 40007; doi: 10.1038/srep40007 (2017).

**Publisher's note:** Springer Nature remains neutral with regard to jurisdictional claims in published maps and institutional affiliations.

## Figures and Tables

**Table 1:** Demographic and clinical details of the study sample.

Parameter	
N	28
Gender [male:female]	11:17
Age [years]	29.4 ± 6.3
Height [cm]	171.7 ± 8.7
Pain Catastrophizing Score	11.9 ± 7.7
Neutral unpleasantness [yes:no]	0:28
Neutral pain [yes:no]	0:28
Cold unpleasantness [yes:no]	5:23
*Rating* [*NRS*]*	3.2 ± 2.8
Cold pain [yes:no]	1:27
*Rating* [*NRS*]*	2 ± 0
Warm unpleasantness [yes:no]	4:24
*Rating* [*NRS*]*	1 ± 0
Warm pain [yes:no]	1:27
*Rating* [*NRS*]*	1 ± 0
TGI unpleasantness [yes:no]	13:15
*Rating* [*NRS*]*	3.7 ± 2.1
TGI pain [yes:no]	3:25
*Rating* [*NRS*]*	3.7 ± 2.5

^*^Rating was only given by those individuals who indicated unpleasantness or pain, respectively.

NRS: Numeric rating scale (0 = no pain, 10 = most bearable pain).

TGI: Thermal Grill Illusion (interlaced application of cold and warm).

Results are displayed as mean ± standard deviation.

**Table 2:** Contact heat evoked potentials: Summary of N2 and P2 latencies, N2P2 amplitude, N1 latency, N1 amplitude, and pain rating.

Group	Time-point of Stimulation	
**Cold**	**Neutral 32 °C**	**Cold 20 °C**
N2 Latency [ms]	267.5 ± 24.1	268.2 ± 26.3
P2 Latency [ms]	394.8 ± 51.7	391.8 ± 45.4
N2P2 Amplitude [μV]	34.5 ± 12.9	30.1 ± 11.7
Pain rating (NRS)	4.2 ± 2.7	4.0 ± 1.7
N1 Latency [ms]	210.9 ± 48.8	214.3 ± 31.5
N1 Amplitude [μV]	−7.8 ± 3.1	−6.2 ± 3.5
**Warm**	**Neutral 32 °C**	**Warm 40 °C**
N2 Latency [ms]	275.8 ± 26.7	275.8 ± 11.3
P2 Latency [ms]	393.63 ± 38.8	397.5 ± 35.1
N2P2 Amplitude [μV]	33.3 ± 12.1	28.9 ± 11.3
Pain rating (NRS)	3.5 ± 1.6	3.5 ± 1.6
N1 Latency [ms]	231.38 ± 26.4	219.7 ± 23.6
N1 Amplitude [μV]	−6.8 ± 2.6	−6.9 ± 3.1
**TGI**	**Neutral 32 °C**	**TGI 20/40 °C**
N2 Latency [ms]	273.2 ± 36.3	267.9 ± 25.5
P2 Latency [ms]	388.2 ± 45.0	384.6 ± 42.4
N2P2 Amplitude [μV]	33.0 ± 12.7	36.2 ± 13.6
Pain rating (NRS)	4.0 ± 1.6	4.3 ± 1.8
N1 Latency [ms]	222.0 ± 20.5	223.3 ± 24.5
N1 Amplitude [μV]	−6.8 ± 1.8	−8.0 ± 2.6

Results are displayed as mean ± standard deviation.

^‡^Bonferroni corrected.
